# Peer Review of “Predicting Escalation of Care for Childhood Pneumonia Using Machine Learning: Retrospective Analysis and Model Development”

**DOI:** 10.2196/71100

**Published:** 2025-03-04

**Authors:** Colin Rogerson

**Affiliations:** 1 Division of Pediatric Critical Care, Regenstrief Center for Biomedical Informatics, Indiana University School of Medicine, 705 Riley Hospital Drive, Indianapolis, IN, United States, 1 (317) 944-7065

**Keywords:** childhood pneumonia, community-acquired pneumonia, machine learning, clinical decision support system, prognostic care decision

*This is the peer-review report for “Predicting Escalation of Care for Childhood Pneumonia Using Machine Learning: Retrospective Analysis and Model Development.”*

## Round 1 Review

### General Comments

The authors [1] have examined the medical records for 437 patients with pneumonia and created a machine learning–based classifier to determine which patients required transfer to a tertiary care center. This subject is interesting, as the predictive power of these novel statistical techniques is high and could improve the clinical care of these patients. The authors have done thorough work describing the statistical methods used in the preprocessing of the data and model development. My primary concerns in the manuscript are the lack of clinical application description, the lack of description of the time frame of the included data elements, and the lack of description regarding the patient population and outcome of interest. The following are my point-by-point comments.

### Specific Comments

#### Major Comments

##### Abstract

- The authors use the term “case management” in the Abstract and several times in the manuscript. In this context, the authors’ meaning is the decision for the escalation of care or patient transfer. However, in US-based hospital systems, case management has a different meaning, which includes largely transition to rehabilitation or nursing facilities, acquisition of home oxygen therapy, etc. I would recommend altering this term for comprehension to something like “escalation of care” or “patient triage.”
- The primary outcome of interest should be included in the Abstract.
- As detailed in the Methods section, it is crucial to describe the time frame for the included variables, to know when the algorithm could be used in clinical practice.

##### Introduction

- As the goal of the algorithm in the study is to predict which patients will need transfer to tertiary care for increasing respiratory support, more of the Introduction should focus on the management of in-hospital pediatric pneumonia, challenges, and reasons for the escalation of care.
- I would recommend altering the sentence that describes pneumonia as easily preventable and treatable. Several of the most complicated cases in the intensive care unit are admitted with pneumonia.

##### Methods

- While great care is taken to describe the approach to data preprocessing, feature selection, and model development, I would recommend following the TRIPOD (Transparent Reporting of a Multivariable Prediction Model for individual Prognosis or Diagnosis) guidelines [2], which are validated reporting recommendations for predictive models.
- Please provide more details regarding the hospital systems involved in this study. Are they large, academic centers or small, rural centers?
- For study inclusion, I am not familiar with the Integrated Management of Childhood Illness guidelines. Are these structured diagnostic codes captured in the electronic health record? Is it a computational phenotype?
- Please specify what is meant by “neonatal age.”
- Many of the variables included in the model are colinear. For example, age and weight are highly dependent on one another, and including both in the model can be detrimental. The feature selection methods may be able to discern this, but maybe not. I would recommend using only age and *z* score in the model.
- The time frames are not stated for the variables. For example, does “hypoxia” mean hypoxia at any time during the hospitalization? On hospital admission? In the first 12 hours? This information is vital to determine the usability of the entire model. If the model uses variables available during the entire hospitalization, the predictive ability will be high, but the usability will be low. A model that can predict right when a patient is transferred to a tertiary care center that the patient will be transferred is useless. However, a model that can predict on admission, or in the first 6‐12 hours, that a patient will require transfer is incredibly helpful. Without knowing the time frame for these variables, we cannot assess how the model could be applied in clinical practice.
- Please provide clarity regarding the study outcomes. The primary outcome is described as whether the patient was referred to a tertiary care center or not. The next sentence describes “poor prognosis” as pediatric intensive care unit admission or oxygen/ventilation support. How is this outcome used? Is this a secondary outcome? Is this describing the reason for transfer? Please clarify.
- As stated in the TRIPOD guidelines, you should present the amount of missingness in your data. It appears you used imputation methods for missing data. It is helpful to describe the amount of missing data that was imputed and the method for imputation.

##### Results

- There is a glaring lack of information regarding your study population. Please provide a table describing patient characteristics including demographics and the variables you used in the algorithm. Also, please provide a comparison between the patients who were transferred to a tertiary care center and those who were not.
- In imbalanced datasets, it can be more useful to measure model performance using the area under the precision-recall curve rather than the standard area under the receiver operator characteristic curve. I would recommend adding this metric.

##### Discussion

- The Discussion, overall, focuses much more on the technical details of the data curation and model development than it does on the clinical application of the model. Much of the technical details presented are also clearly explained in the Methods section and then repeated in the Discussion. I would recommend substantial revision to the Discussion section to remove redundant information that is already contained in the Methods section, as well as the addition of how this model could be applied in a clinical setting to improve the care of patients with pneumonia.
- The Discussion contains no information regarding the limitations of the study. Please describe in detail the prominent limitations of the study. These should include the use of retrospective data, including only two centers, imbalanced data, challenges with clinical implementation of the model, etc.
- The Discussion, and other areas of the manuscript, mention disease prevention several times. The goal of this study has nothing to do with the prevention of pneumonia, only the treatment of pneumonia and the prevention of associated morbidity and mortality. Please revise.

##### Conclusion

- As it stands, the Conclusion is fairly long and does not focus only on the primary findings of the study. I would recommend trimming it to 2‐3 sentences that focus only on the primary findings of the study, such as the feasibility of developing this type of predictive model and the potential applications of the model to clinical practice.

### Minor Comments

#### Methods

- The authors describe that ensemble methods “significantly enhance the accuracy of classifications.” Please provide a reference for this statement.

#### Results

- Please provide numbers for those who met your primary outcome of interest (transfer to a tertiary care center).
- Please provide a description of the time frame for patient transfer, for those who were transferred.

#### Discussion

- It would be interesting to hear more regarding the use of this model in resource-limited settings and the benefits it could provide.

## Round 2 Review

### General Comments

The authors have conducted a single-center, retrospective study evaluating the derivation and performance of a machine learning model to predict the need for transfer to a higher level of care for childhood pneumonia. The authors were provided with a substantial amount of feedback on the original submission, and although the authors’ response is detailed and comments on how all concerns were adequately addressed, the resulting manuscript is lacking in many if not most of the requested changes. The revised manuscript remains confusing to the reader and bereft of some essential elements of standard study reporting, including a basic description of the patient population and details regarding the timing of variable collection and use in the model. Due to this lack of response to the initial reviewer feedback, I am recommending rejection of this manuscript. The following are my point-by-point critiques, many of which are similar to those in my original review.

### Specific Comments

#### Abstract

- First sentence: Please revise it to “Pneumonia is the leading cause of preventable mortality for children under five years of age.”
- Background: The terms “case management” and “disease prevention” are still used in the Abstract. In my initial review, I recommended revising these terms to improve study clarity, and although the authors stated in their response that they replaced these terms, they remain in the Abstract. As it stands, it is not immediately clear to the reader that the purpose of the study was to provide a tool to assist bedside clinicians to determine which patients are likely to require transfer of care to a higher-level facility for pediatric pneumonia.
- Methods: As it stands, it is confusing to the readers what was actually done in the study. It should be very apparent that the authors used a specific list of variables (please provide each in the Abstract) to predict the need for transfer to a larger institution using a specific type of machine learning model (ensemble). In the current version, this is difficult to discern.
- Results: I would be completely clear regarding the outcome your model is predicting. After reading the paper, it is understood that “pneumonia prognosis” and “severity” actually mean required transfer to a higher level of care, but it is unclear in the Abstract. I would explicitly state “predicted transfer to a higher level of care with 77%‐88% accuracy.”

#### Introduction

- Second paragraph, fifth sentence: I would recommend revising it to “However, this preventable health problem continues to be a substantial cause of mortality, especially in underdeveloped countries and regions, due to the lack of equipment and trained human resources.” There is no way to quantify it as “the most important cause of mortality.”
- The term “case management” continues to be used in the Introduction, which decreases clarity for the reader.
- As recommended previously, I would be very specific in the Introduction that you are trying to create a tool to help bedside clinicians (typically non–intensive care physicians) decide when to transfer a patient with pneumonia to a higher level of care to prevent morbidity and mortality. As it stands, this is unclear.

#### Methods

- In my initial review, I asked the authors to clarify what is meant by neonatal age. In their response, they said they had revised the Methods to state specifically 28 days or fewer. However, in the first paragraph of the Methods, it continues to state “neonatal age.” Please revise.
- For clarity, I would recommend restating your primary outcome to simply “required tertiary care referral.” Having the outcome as severe versus nonsevere, which is defined as requiring tertiary care referral or not, adds an extra step to the thought process and can be confusing.
- One of my largest concerns in the initial manuscript was the timing of the variables. This is crucial when determining how useful the model could be. If the elements in Table 1 are measured on admission, or in the first 6‐12 hours of admission, the model could be very useful for patient care. If the elements were measured at any point during the hospitalization, it becomes much less useful. My worry is that the model was developed based on the elements’ presence at any point, meaning if the child had fever, cough, respiratory distress, and hypoxia at hour 48, then at hour 49 the model was able to predict the patient would need transfer, and the patient was transferred at hour 50—this is not helpful to clinicians. On the other hand, if the model predicts at hour 12 that a patient needs transfer, and then at hour 50 they transfer, that is potentially very helpful to clinicians. Without these details, I cannot recommend the publication of the manuscript.
- It appears that the model was developed using the data from all 437 patients, and the results are presented following k-fold cross validation. It is standard practice to derive the model on a subset of the data (typically 70%‐80%) and then to test it on the remainder of the dataset to prevent overfitting and inflation of performance metrics. It does not appear that this was done. Despite having a small sample size, I believe this approach would lead to a more robust and generalizable model.

#### Results

- The first paragraph contains many “nuts and bolts” details of model development, and these would be better positioned in the Methods section.
- Both reviewers on the initial submission requested additional details describing the study population, and although the authors responded that they added these details, there are still none provided. It is essential to the understanding of the study results to know the characteristics of the patient population, and it should be a standard requirement for all clinical studies.
- The Shapley additive explanations value results presented in Figure 2 are valuable, but more details describing each measured factor are required. I recommend a table with each factor as rows and two columns comparing the population that did not require transfer to a tertiary care center to the population that did.
- An additional figure showing an area under the precision-recall curve for each model would also be interesting to the readers.

#### Discussion

- The Discussion spends a decent amount of space discussing the COVID-19 pandemic. While this does have some bearing on the management of childhood pneumonia, I believe the space would be better spent discussing the actual implementation of this type of algorithm. How would a primary care clinician actually use this model in practice? How would it improve upon current clinical practice? Would it be easy or difficult to incorporate into routine workflows? This would be more interesting to the readers.
- I recommend adding what the next steps of this line of research would be. How would you seek to improve the model’s performance? More patient data? Additional variables?
- In the original submission, I recommended the authors provide a limitations section and also provided some examples. Although the authors response says they added this, there are still no limitations provided. Please provide this essential element to the Discussion.

#### Conclusion

- I recommend commenting on what the next steps of this line of research would be in more specific terms.

## Round 3 Review

### General Comments

The authors have conducted a single-center, retrospective study evaluating the derivation and performance of a machine learning model to predict the need for transfer to a higher level of care for childhood pneumonia. The authors were provided with a substantial amount of feedback on the original submission and have been responsive to feedback, which has resulted in a much improved manuscript. There remain several typographical and grammatical errors, which I would advise an English-grammar expert to review prior to publication, but from a scientific standpoint, I believe the manuscript is appropriate for publication.

### Specific Comments

#### Major Comments

1. Details regarding the patient population have been provided in detail.

2. The study objectives have been clarified for readers.

3. The study methods are now much more reproducible.

## Abbreviations

TRIPOD: Transparent Reporting of a Multivariable Prediction Model for individual Prognosis or Diagnosis
